# Weight-loss and exercise for communities with arthritis in North Carolina (we-can): design and rationale of a pragmatic, assessor-blinded, randomized controlled trial

**DOI:** 10.1186/s12891-017-1441-4

**Published:** 2017-02-22

**Authors:** Stephen P. Messier, Leigh F. Callahan, Daniel P. Beavers, Kate Queen, Shannon L. Mihalko, Gary D. Miller, Elena Losina, Jeffrey N. Katz, Richard F. Loeser, Sara A. Quandt, Paul DeVita, David J. Hunter, Mary F. Lyles, Jovita Newman, Betsy Hackney, Joanne M. Jordan

**Affiliations:** 1 J.B. Snow Biomechanics Laboratory, Department of Health & Exercise Science, Wake Forest University, Winston-Salem, NC 27109 USA; 2 Thurston Arthritis Research Center, University of North Carolina at Chapel Hill, Chapel Hill, NC USA; 3 Department of Biostatistical Sciences, Wake Forest School of Medicine, Winston-Salem, NC USA; 4 Haywood Regional Medical Center, Clyde, NC USA; 5 Orthopedic and Arthritis Center for Outcomes Research, Department of Orthopedic Surgery, Brigham and Women’s Hospital, Harvard Medical School, Boston, MA USA; 6 Department of Epidemiology and Prevention, Wake Forest School of Medicine, Winston-Salem, NC USA; 7 Department of Kinesiology, East Carolina University, Greenville, NC USA; 8 Rheumatology Department, Institute of Bone and Joint Research, Kolling Institute, University of Sydney, Sydney, Australia; 9 Section on Gerontology and Geriatric Medicine, Wake Forest School of Medicine, Winston-Salem, NC USA; 10 Department of Rheumatology and Immunology, Wake Forest School of Medicine, Winston-Salem, NC USA

**Keywords:** Osteoarthritis, Knee pain, Clinical trial, Pragmatic, Community based research

## Abstract

**Background:**

Recently, we determined that in a rigorously monitored environment an intensive diet-induced weight loss of 10% combined with exercise was significantly more effective at reducing pain in men and women with symptomatic knee osteoarthritis (OA) than either intervention alone. Compared to previous long-term weight loss and exercise trials of knee OA, our intensive diet-induced weight loss and exercise intervention was twice as effective at reducing pain intensity. Whether these results can be generalized to less intensively monitored cohorts is unknown. Thus, the policy relevant and clinically important question is: Can we adapt this successful solution to a pervasive public health problem in real-world clinical and community settings? This study aims to develop a systematic, practical, cost-effective diet-induced weight loss and exercise intervention implemented in community settings and to determine its effectiveness in reducing pain and improving other clinical outcomes in persons with knee OA.

**Methods/Design:**

This is a Phase III, pragmatic, assessor-blinded, randomized controlled trial. Participants will include 820 ambulatory, community-dwelling, overweight and obese (BMI ≥ 27 kg/m^2^) men and women aged ≥ 50 years who meet the American College of Rheumatology clinical criteria for knee OA. The primary aim is to determine whether a community-based 18-month diet-induced weight loss and exercise intervention based on social cognitive theory and implemented in three North Carolina counties with diverse residential (from urban to rural) and socioeconomic composition significantly decreases knee pain in overweight and obese adults with knee OA relative to a nutrition and health attention control group. Secondary aims will determine whether this intervention improves self-reported function, health-related quality of life, mobility, and is cost-effective.

**Discussion:**

Many physicians who treat people with knee OA have no practical means to implement weight loss and exercise treatments as recommended by numerous OA treatment guidelines. This study will establish the effectiveness of a community program that will serve as a blueprint and exemplar for clinicians and public health officials in urban and rural communities to implement a diet-induced weight loss and exercise program designed to reduce knee pain and improve other clinical outcomes in overweight and obese adults with knee OA.

**Trial registration:**

clinicaltrials.gov Identifier: NCT02577549 October 12, 2015.

## Background

Worldwide, only half of the population seeking osteoarthritis (OA) health management receive appropriate care. This is due, in part, to limited prescription of non-pharmacologic interventions, the rise of pharmacologic prescriptions for symptom management, low patient health literacy, and barriers impeding lifestyle modifications [1, 2]. Knee OA is the most common and persistent cause of mobility dependency and disability; its prevalence is estimated at over 250 million, or 3.6% of the world’s population [3, 4]. OA is chronic – patients live with the symptoms for an average of 26 years [5]. With no effective disease-modifying interventions, treatment focuses on pain relief, but the safety concerns associated with many pain medications highlight the need for safe, effective non-pharmacologic interventions. Clinical guidelines strongly encourage the use of non-pharmacologic exercise and diet to relieve pain and improve function [6, 7]. Unfortunately, only 25% of the patients requiring advice concerning these treatments (BMI ≥ 27 kg/m^2^ with diagnosed knee OA) receive the necessary care [8]. Moreover, these interventions, proven effective under highly controlled conditions, have yet to show long-term improvements in community settings [9–11].

**Fig. 1:**
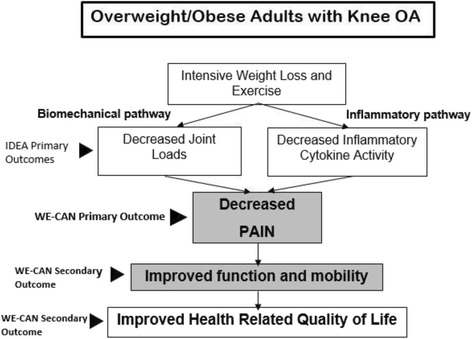
Our model developed for IDEA and used for **we-can** indicating the pathways by which intensive weight loss and exercise can decrease joint loads and inflammation leading to decreased pain and improved mobility and health related quality of life

The association between obesity and knee OA was first documented in 1945 and has been widely verified [12–15]. Two in three obese people may develop symptomatic knee OA in their lifetime [16]. Obesity is the most modifiable risk factor for knee OA, and weight loss is a safe, effective non-pharmacologic intervention to improve clinical outcomes [9, 10]. Recently, our Intensive Diet and Exercise for Arthritis (IDEA) trial compared a diet-induced weight-loss (D) of 10% of baseline weight and exercise (E) interventions, separately and in combination (D + E), across an 18-month period in 454 overweight and obese older adults with knee OA. The primary mechanistic outcomes, knee joint loads and inflammation, were reduced in D and D + E compared to E [10]. An intent-to-treat analysis also revealed that while D and E successfully reduced pain after 18 months (D: 25%, E: 28%), the combination of D + E was twice as effective with a 51% decrease from baseline. The D + E group was also superior to the E group on other clinical measures including physical function and walking speed. IDEA, with its 10% weight loss, produced superior results to our previous trial (ADAPT) of exercise and 5% weight loss that resulted in a 30% reduction in pain [9]. Taken together, these trials provide strong support for our model (Fig. 1) indicating that weight loss plus exercise improves clinical outcomes by affecting both the biomechanical and inflammatory OA disease pathways, decreasing knee joint loads and pro-inflammatory cytokine activity.

IDEA was an efficacy study implemented in a university setting under rigorously monitored conditions that included highly educated and well trained staff, and excellent, well-maintained facilities. Clinicians have expressed concern about the lack of practical means to implement and sustain a similar program in a community-based environment. Thus, determining whether we can adapt this successful program to a pervasive public health problem in real-world clinical and community settings is a clinically important question that has public policy implications.

Approximately 25% of the United States population lives in rural communities [17–19] with most having fewer services and resources than urban communities [20]. Rural dwellers report poorer health-related quality of life [21], reflecting higher prevalence of many disorders, including knee OA. Delivering proven health interventions to communities with limited healthcare access is a national public health priority [22]. Weight-loss and Exercise for Communities with Arthritis in North Carolina (**we-can**) is the first long-term trial of diet-induced weight loss and exercise in older adults with knee OA implemented under pragmatic conditions in both rural and urban communities. Building on the results of IDEA, the intent of the **we-can** trial is to translate this highly beneficial, long-term intervention for a major chronic health condition to diverse community settings.

Few trials are purely pragmatic; most fall on a continuum between pragmatism and efficacy [23]. The pragmatic components of **we-can** include: (1) large sample size (N = 820); (2) broad inclusion criteria; (3) patient-centered outcomes; (4) conducted in established community facilities rather than a referral or university center; (5) commonly available community-based staff to deliver the standardized treatment; (6) non-physicians trained by our physicians to perform a knee exam based on American College of Rheumatology (ACR) clinical criteria; and (7) diverse means of communication with participants (e.g., phone, email, study website, Facebook, text messages). Our experience with ADAPT [9] and IDEA [10] indicates participants need regular interaction with study staff to achieve significant weight loss. Hence, the primary efficacy component of our design is the use of diverse methods of contact by staff who are not physicians but trained community interventionists to maintain participant interaction. The pragmatic nature of many aspects of this trial combined with cost effective methods of staff-patient communication is more likely to improve health care that can be generalized and applied in varied community settings [24].

## Methods/Design

### Trial organization

**Fig. 2:**
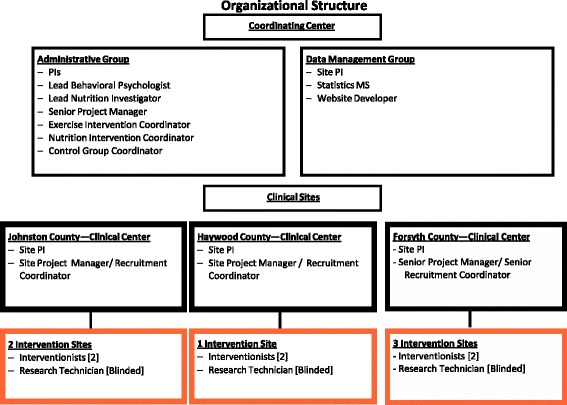
**we-can** organizational structure

The intervention is delivered in communities across North Carolina including 3 sites in Forsyth County, 2 sites in Johnston County, and 1 site in Haywood County. The Coordinating Center at Wake Forest University and Wake Forest Health Sciences in Forsyth County consists of the Administrative and Data Management Groups that oversees day-to-day operations, including recruitment, randomization, data uploads to a central, secured web site, training sessions, coordinating central resources, reporting to the Data Safety and Monitoring Board (DSMB), and collaborating on manuscripts describing trial results (Fig. 2). The Executive Committee (Principal Investigators (PIs), site PIs, ad hoc member), with input from the co-investigators, is responsible for major policy decisions governing trial conduct. Other standing committees include: a Community Advisory Board that consists of a physician and patient advocate from each county; Recruitment; Nutrition and Exercise Intervention; Nutrition and Health Attention Control; Adherence and Retention; Safety; Cost-Effectiveness; Intellectual Property; Ancillary Studies; and Publications and Presentations.

## Research design and methods

### Study design

**we-can** is a Phase III, two arm parallel, pragmatic, assessor-blinded, community-based trial examining the effects of an intensive diet-induced weight-loss and exercise intervention under real-world conditions. It focuses on outcomes that matter to patients, such as reduced pain (primary outcome) and improved function, mobility, and health-related quality of life. It uses broad inclusion criteria, community facilities, and community exercise leaders and nutrition advisors to deliver interventions. Strategies to maintain or improve adherence are based in social cognitive theory and are tailored to real-world delivery.

### Study sample

Participants will include 820 ambulatory, community-dwelling, overweight and obese (BMI ≥ 27 kg/m^2^) men and women who meet the ACR clinical criteria for knee OA of age ≥ 50 years, knee pain on most days of the week, plus at least 2 of the following: stiffness < 30 min/day; crepitus; bony tenderness; bony enlargement; no palpable warmth [25]. With few exclusion criteria (Table 1), participants with self-reported disability due to knee OA are randomized into diet-induced weight-loss and exercise (D + E) or nutrition and health attention control (N + H) groups, regardless of anticipated risk, past compliance, or comorbidities. All participants may maintain their regular medications including analgesics. If pain decreases, they may reduce them with their physician’s consent. Medications are recorded at baseline, 6-, 12-, and 18-month follow-up visits. The study protocol was reviewed and approved by the Human Subjects Committees of Wake Forest Health Sciences (Human protocol: IRB33618) and The University of North Carolina at Chapel Hill (Human Protocol: IRB15-1427) and is in compliance with the terms and conditions set forth in the Helsinki Declaration.

**Table 1:** Exclusion criteria

Criteria	Exclusion	Method
Significant co-morbid disease that would pose a safety threat or impair ability to participate; low BMI; no knee OA	Symptomatic or severe coronary artery disease; unable to walk without assistive device; blindness; type 1 diabetes; BMI < 27.0 kg/m^2^, does not meet ACR clinical criteria for knee OA	Medical history; knee exam using ACR clinical criteria and performed by research staff trained by our MDs
Ability and willingness to modify dietary or exercise behaviors	Unwillingness or inability to change eating and physical activity habits due to environment; cannot speak and read English	Questionnaire, assessment by interventionists
Ability to finish or to comply with the 18-month study	Planning to leave area ≥ 2 months during the next 18 months	Questionnaire
Significant cognitive impairment	Montreal Cognitive Assessment (MOCA)	Medical history, MOCA
Transportation	Unable to provide own transportation to and from the intervention site	Questionnaire

### Randomization

Each eligible participant is randomized to one of the two arms of the clinical trial (D + E or N + H) according to a variable block-length algorithm that is controlled by the Data Management Group. Randomization is stratified by intervention site (i.e., Forsyth, Haywood, Johnston counties) to ensure balance for the two interventions with respect to each site’s population characteristics (e.g., residential, socioeconomic). Within each county, participants are also stratified based on BMI (27.0-34.9, ≥ 35.0 kg/m^2^) and gender to ensure balance across these factors. Randomization uses a web-based system that is part of the study data management system.

### Clinical sites

Table 2 describes each community intervention site. Johnston County is non-metropolitan with small cities and rural areas; Forsyth County is metropolitan, with the city of Winston-Salem and surrounding suburbs; Haywood County is non-metropolitan with rural and small city areas. To maintain assessor blinding, each county has a site to collect participant outcome data that is separate from the intervention sites.

**Table 2:** Description of intervention sites by county

County	Site Name	Description	Size (sq.ft)	Exercise	Nutrition
Forsyth population 361,220 859 persons/mi^2^	Healthy Exercise Lifestyle Programs	Community, university setting	20,000	track, aerobic equipment strength equipment	2 classrooms
	Smiley Fitness	Private	2,200	aerobic, strength equip.	1 classroom
	St. Peter’s World Outreach Center	Church	73,000	track, strength equipment.	2 classrooms with kitchen
Johnston population 177,967 213 persons/mi^2^	Johnston Health Care Medical Mall	Facility within Medical Mall affiliated with Hospital	197,000	Track, strength equipment	1 classrooms
	Clayton Community Center	Parks/Rec Center	34,000	Track, aerobic equipment strength equipment	2 classrooms
Haywood population 59,183 106 persons/mi^2^	MedWest Fitness Center	Hospital Fitness Center	54,000	Track, aerobic equipment strength equipment	2 classrooms

### Interventions

The goal of the D + E intervention is a loss of at least 10% of baseline body weight as recommended by the National Institutes of Health [26] for overweight and obese adults and is consistent with our results in IDEA in which an 11.4% weight loss combined with exercise reduced knee pain by 51% [27]. The N + H attention control group, modeled on similar groups in our previous studies, provides attention, social interaction, and nutrition and health education [28, 29].

#### Intensive diet-induced weight loss

The dietary plan is characterized by frequency of contacts, methods to induce caloric restriction, and behavioral strategies. For the first 6 months, the plan is based on an energy-restricted diet using 1 to 2 partial meal replacements (Lean Shakes, GNC®) per day provided by the study with the option to incorporate 1 meal replacement per day during months 7–18. The plan is individualized and based on the program used in IDEA [30]. Based on IDEA, most participants will reach their weight-loss goal after 9 months. Once it is achieved, they may self-select to begin weight maintenance or continue to lose weight using safe, healthy nutrition practices, provided the participant is motivated to continue losing weight and has not reached a level associated with possible health hazards; i.e. > 20% body weight loss at 6 months or >30% at 12 months [31, 32].

Initial diet plans ensure an energy-intake deficit of 800–1000 kcals/day from the estimated energy expenditure (predicted resting metabolism calculated using the Owen equation [33] x 1.2 activity factor). The exercise program should expend an average of 200 kcals/day, for a total imbalance of at least 1,000 kcals/day. The lowest intake will be 1,100 kcals for women and 1,200 kcals for men. The distribution goal will be 15–20% protein with at least 1.2 g protein/kg ideal body weight; <30% fat; <10% saturated fatty acids; and 45–60% carbohydrates. These levels are consistent with the Dietary Reference Intakes for Energy and Macronutrients and successful weight-loss programs [34]. The amount of protein is based on recent evidence showing a greater need for older adults to support good health [35]. The NIH Diet History Questionnaire is used to assess dietary intake [36, 37].

There is flexibility in the intervention that permits participants to utilize other forms of healthy, low-calorie meal replacements or pre-portioned meals to replace the Lean Shakes. For months 7–18, participants consume meals with healthy foods that follow a recommended diet plan with an option to incorporate 1 meal replacement per day. Meals are targeted to contain 400–600 kcals and low in fat and sugars and high in vegetables, fruits, and whole grains. Snacks (~100-120 kcals) may include a nutrition bar, fruit, or vegetable. Daily energy intake is adjusted to the rate of weight change with a goal of 1 kg lost per wk. Each participant is encouraged to take a daily multivitamin/mineral supplement containing up to 100% of the Dietary Reference Intake per nutrient. Intervention staff assists in developing meal plans to provide the prescribed macronutrient-balanced energy intake.

The intervention staff conducts group and individual sessions throughout the 18 months (Table 3). Content emphasizes nutrition and behavioral strategies to attain the weight-loss goal. At the outset, participants select their preferred mode of contact (e.g., phone, email, study website, Facebook, text messages), and information is distributed accordingly. Individual sessions alternate between face-to-face and other modes; group sessions are face-to-face.

**Table 3:** Summary of diet-induced weight loss plan and number of planned contacts

Months	Weight loss plan	Meal Replacements or equivalent per day (N)	Contacts per month (N)		
			Total	Individual	Group
0-6	Energy restriction 800–1000 kcals/day	1-2	4	2^a^	2
7-12	Either continued energy restriction or weight maintenance once 10% weight loss reached	0-1	2	1^a^	1
13-18	Either continued energy restriction or weight maintenance once 10% weight loss reached	0-1	1	1^a^	none

^a^Individual contacts will alternate between face-to-face and a method preferred by the participant (i.e., phone, email, text, etc.)

#### Alert values

Weight is monitored by the interventionists at each face-to-face meeting. A >20% loss after 6 months or >30% after 12 months triggers a safety alert. This information is shared promptly with the site medical director and the Nutrition and Exercise Committee who will determine the next course of action. This information is reported to the Data and Safety Monitoring Board. Acute adverse effects are rare in a planned weight-loss strategy such as **we-can**, even in older adults. In participants who experience a serious adverse event, the medical director and the participant’s primary care physician consult to determine the possibility of continued study participation.

#### Exercise

The exercise component includes 60-min sessions 3 days per week for 18 months at one of the designated community facilities. The prescribed exercise program consists of aerobic (15 min), resistance-training (20 min), a second aerobic (15 min), and cool-down (10 min) phases. Strength training is particularly relevant to offset any loss of muscle and bone mass resulting from weight loss. In addition to the 3 scheduled days, participants are encouraged to exercise most other days of the week on their own. Our protocol is consistent with the American College of Sports Medicine (ACSM) guidelines for exercise for older adults [38]. Monthly exercise logs are used to monitor progress. Prior to planned absences (e.g., vacations, caregiver duties) participants receive exercise logs and personal instruction on substituting home-based exercises to perform while away.

Walking is the primary mode of aerobic training. A target heart rate range will be calculated as 40–60% of the heart rate reserve using the Karvonen formula below [39]. The range of 40–60% (i.e., fractional intensity) meets the ACSM definition of moderate intensity exercise.

$$ \mathrm{Target}\ \mathrm{Heart}\ \mathrm{Rate}\ \mathrm{Range} = \left(\mathrm{fractional}\ \mathrm{intensity}\right)\left({\mathrm{HR}}_{\max } - {\mathrm{HR}}_{\mathrm{rest}}\right) + {\mathrm{HR}}_{\mathrm{rest}} $$

$$ \mathrm{Where}\ {\mathrm{HR}}_{\max } = \mathrm{age}-\mathrm{predicted}\ \mathrm{maximum}\ \mathrm{heart}\ \mathrm{rate},\ \mathrm{using}\ 208-0.7\ \left(\mathrm{age}\right) $$

$$ {\mathrm{HR}}_{\mathrm{rest}} = \mathrm{resting}\ \mathrm{heart}\ \mathrm{rate} $$

The goal of the strength training is to improve mobility and balance and attenuate loss of muscle and bone mass. Resistance exercises include hip abduction/adduction, leg extension, leg curl, leg press, and heel raise/calf press. Depending on the resources available at each community facility, machines, Thera-bands™, free weights, or the participants’ own body weight are used. Two sets of 12 repetitions of each exercise are performed with a 1–1.5 min rest between sets. Home exercise serves as a backup for days when facility based participation is not possible; participants are provided Thera-bands™ and a manual to continue the same regimen as in the facility.

#### Nutrition and health group

One half of the participants (N = 410) are randomized to the Nutrition and Health attention control group, modeled after our FAST [40], ADAPT [9], and START [28] control groups. It provides attention, social interaction, and evidence-based nutrition and health education delivered in 1-h, face-to-face group meetings during months 1, 3, 6, 9, and 15, and via informational packets and webinars during alternate months. The sessions are interactive and provide useful information on such topics as protein and dairy, food labels, organic and non-organic foods, holiday eating, and stress management. In addition, N + H group members receive 100 USD for completing their testing visits; 25 USD at 6 month testing and 75 USD at 18 month testing. The face-to-face meetings feature a community health professional, a member of the Community Advisory Board, or a **we-can** investigator.

#### Techniques to improve adherence and retention

**we-can’s** design evolved from Social Cognitive Theory (SCT), group dynamics, and our experience with weight management and exercise trials. SCT suggests that behavior is learned through on-going interactions among the person, environment, and the behavior; behavior change is complex and people don’t simply react to the environment. For example, nutrition practices (behavior) in overweight and obese older adults with knee OA may be affected by the amount of pain experienced (person) and by interactions with interventionists in the community setting (environment). The study behavioral psychologist trains **we-can** community interventionists in standardized and validated behavioral techniques based on a SCT framework to enhance adherence. They include regular contact using diverse methods; positive feedback; establishing personal commitment to the project; promoting a sense of community via study logo, cards, and newsletters; and developing self-efficacy, realistic outcome expectations, and self-regulatory skills. The importance of regular attendance is emphasized, and data reviewed regularly to identify those who need additional reminders and strategies from the toolbox.

Our toolbox approach tailors the intervention to each participant. When a problem or barrier to participation is identified, a strategy is tested for a specific period of time to overcome that problem. If the problem is resolved, the strategy is continued until behavior change is consistent. If not, a new strategy is selected and tested for a specific period. For example, if a participant develops a caregiver conflict with their scheduled group exercise session, an alternate time is identified to improve attendance; if that new time still doesn’t work then options to exercise at home are provided. Communication is scheduled regularly via their preferred mode of contact to discuss progress and home logs are monitored over time to ensure that this new strategy is successful. Some of these strategies are used in groups, while others require one-on-one interaction via the participant’s preferred mode of contact. The Adherence and Retention Committee reviews adherence rates at regular intervals; discussions focus on site-specific adherence rates, barriers to participation, and strategies to promote adherence.

#### Training study personnel

Coordinating Center personnel provide on-site training for the community interventionists during the 9-month planning period (and during the course of the trial for newly hired interventionists) and tailor the instruction to the local facilities. For example, some exercise facilities have indoor tracks conducive to walking, while others with less space use treadmills, elliptical trainers, stationary bikes, or low-impact aerobic dance. Some facilities have a full kitchen for nutrition classes, while others have no kitchen facilities in meeting locations. Tailoring the intervention to each facility and employing and training people from the local community supports our pragmatic study design. Following the initial training sessions, the Coordinating Center’s interventionist team monitors fidelity and the progress of each site via bi-weekly WebEx meetings with our psychologist, examining adherence rates, barriers to participation, noncompliant participants, and strategies that have proved successful. These core values are consistent, effective processes for translating knowledge into practice [41].

#### Protocol deviations

Study staff reports all protocol deviations on the study website. Protocol deviations that do not constitute an unanticipated problem are submitted to the Safety Committee on a monthly basis for quarterly review by the Coordinating Center. Any protocol deviation that results in an unanticipated problem and impacts participant safety is reported to the Safety Committee within 24 h and to the Institutional Review Board (IRB) and National Institute of Arthritis and Musculoskeletal and Skin Disease (NIAMS) via KAI (NIAMS subcontractor) within 48 h of the event. KAI staff distribute these reports to the DSMB.

#### Adverse Event (AE) collection and reporting

Participants are queried monthly regarding AEs and encouraged to report AEs to their interventionist soon after they occur. These include any new events not present during the pre-intervention period or events that were present during the pre-intervention period that have increased in severity. Some delayed onset muscle soreness (DOMS) is expected after training sessions; excessive DOMS could be reported as an AE, depending on severity. SAEs are reported to the site PI and site physician within 24 h of notification by the participant. Non-serious adverse events (related and unrelated to the study) are reported to the site PI and site physician within 7 days of notification of the event; the site PI and study physician will report serious adverse events (SAE) to the Safety Committee within 24 h and to NIAMS via KAI within 48 h of the initial report (Table 4).

**Table 4:** Reporting of serious adverse events

Type of Event	Reporting Requirements	Reporting Requirements	Reporting Requirements
	Initial Report	Clinical Site	Safety Committee
Serious Adverse Events (SAE)	Reported to Site PI/Physician immediately	Within 24 h of initial receipt of the information Clinical Site will report to Safety Committee	Within 24 h of initial receipt of the information Safety Committee will report to NIAMS (48 h from initial report)
Non serious Adverse Events (AE)	Reported to Site PI/Physician within 7 days	Reported in monthly correspondence to Safety Committee	Reported in Bi-annual correspondence to NIAMS

### Trial conduct

#### Recruitment

Recruitment goals for each county were based on previous recruitment success and population density; Forsyth, Johnston, and Haywood Counties’ recruitment goals are 450, 220, and 150, respectively (total = 820). With fewer exclusion criteria than in our previous trials, we conservatively estimate an average yield of 15%. A web-based data tracking system monitors recruitment strategies in each county.

We use overlapping recruitment strategies and a monitoring system that provides feedback regarding the effectiveness and cost of each. Forsyth County uses mailings, local newspaper ads, and Wake ONE, the Wake Forest Baptist Health patient database. We also have strong ties with local aging service networks and access to senior centers, senior high-rise residential sites, churches, and a large database of older adults who have signed consent to be contacted about participating in future clinical trials. The University of North Carolina Chapel Hill has a similar relationship with Johnston County and has ready access to a large segment of the population, many who have signed consent to be contacted for future studies. The most successful recruitment methods for clinical trials in the county have been through the Johnston County Health System (which is part of the UNC Health System and patient database), primary care offices, local newspapers, radio/TV shows, the Seniors Guide, a Parks and Recreation Brochure, the newsletter for the Town of Clayton, and the Clayton Community Center. Methods of recruitment in Haywood include community talks, physician practices, local newspaper articles and/or advertisements, local orthopedic groups, and the Haywood Health and Fitness Center.

The three recruitment coordinators plan strategies and activities for each clinical site. Our experience indicates that monitoring the recruitment process continually is necessary to achieve study goals. At bi-weekly meetings, the Recruitment Committee reviews all recruitment activities, plans new ones, and monitors the number of phone contacts, screening visits (SV1), and participants randomized. We estimate yields as the project progresses to determine whether our strategies should be intensified or altered. Specific strategies aim to maximize the number of African-Americans who qualify for, and are enrolled in the study. Haywood and Johnston counties contribute heavily to our rural population, another underrepresented cohort in clinical trials.

#### Timeline

Recruitment for each of 10 waves takes approximately 3 months with an average of 82 participants/wave across all three intervention sites; an average of 45, 22, and 15 participants are randomized per wave for Forsyth, Johnston, and Haywood counties respectively (Table 5).

**Table 5:** Study time line with milestones

Planning Months1-6	1^st^ subject enrolled in intervention		25% enrolled		50% enrolled		75% enrolled		100% enrolled	
	Wave 1	Wave 2	Wave 3	Wave 4	Wave 5	Wave 6	Wave 7	Wave 8	Wave 9	Wave 10
Recruitment	months 7-9	10-12	13-15	16-18	19-21	22-24	25-27	28-30	31-33	34-36
Intervention	10-27	13-30	16-33	19-36	22-39	25-42	28-45	31-48	34-51	37-54
N	82	164	246	328	410	492	574	656	738	820
Analysis and Final Report: Months 54-60										

### Measurements

#### Screening and follow-up visits

**Fig. 3:**
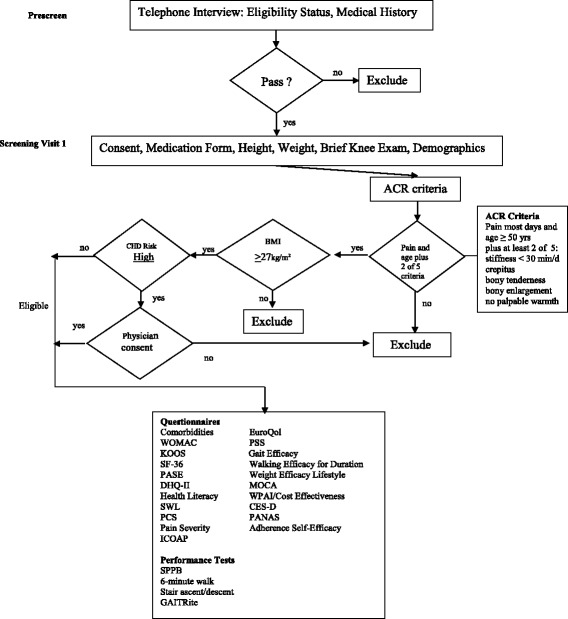
Participant eligibility and screening flow chart. BMI = body mass index; CHD = cardiovascular heart disease; ACR = American College of Rheumatology

The Western Ontario McMasters Universities Osteoarthritis Index (WOMAC) pain (primary outcome) and function (secondary outcome) subscales are detailed below [42, 43]. Other secondary measures including the appropriate citations that detail the metrics for each test instrument are listed in Table 6. Questionnaires and performance measures are completed at baseline, 6 month follow-up (FU6), FU12, and FU18. Participant eligibility and screening flow chart are shown in Fig. 3.

**Table 6:** Measurements with screening and follow-up visit schedule

Measurements	PSV	SV1	FU6	FU12	FU18	Explanation
Questionnaires						
Informed Consent		x				
Eligibility Questionnaire	x					To determine eligibility
Medical History	xc	x	x	x	x	For eligibility and to document changes in health
Risk Stratification	xc	x				Used to screen for cardiovascular risk [54]
Comorbidities Questionnaire		x	x	x	x	[55]
Randomization		x				
WOMAC		x	x	x	x	Pain is primary outcome [42]
KOOS		x	x	x	x	Pain, symptoms, ADL, sport, recreation, knee QOL [56]
ICOAP		x	x	x	x	Intermittent and constant osteoarthritis pain, 2 sleep questions [57]
Cost Effectiveness		x	x	x	x	
PASE scale		x	x	x	x	Physical Activity Scale for the Elderly [58]
MOCA		x			x	Montreal Cognitive Assessment [59, 60]
CES-D		x	x	x	x	The Center for Epidemiologic Studies Depression Scale [61]
EuroQol Quality of Life(EQ5D)		x	x	x	x	Quality of life measure [62]
Work History Resource		x	x	x	x	Visits to clinicians, tests, medications, injections, surgery, alternative therapies
Work Productivity and Activity Impairment Index		x	x	x	x	Assesses absenteeism and presenteeism [63]
DHQ II		x	x	x	x	NIH Diet History Questionnaire [36, 37]
SF-36		x	x	x	x	Health related quality of life [physical, mental] [64]
Self-Efficacy-Adherence		x	x	x	x	Belief can exercise over time [65]
Perceived Stress (PSS)		x	x	x	x	The degree to which people perceive their lives as stressful [66]
Pain Catastrophizing Scale (PCS)		x	x	x	x	Catastrophizing [rumination, magnification, and helplessness] [67, 68]
Healthy Literacy		x	x	x	x	Functional health literacy [69]
Walking Efficacy for Duration		x	x	x	x	Confidence in walking for different durations [65]
Gait Efficacy		x	x	x	x	Confidence in completing gait related tasks [65]
Weight Efficacy Lifestyle Questionnaire (WEL)		x	x	x	x	Self-Efficacy for weight management [70]
Positive and Negative Affect Scale (PANAS)		x	x	x	x	Positive and Negative Affect [71]
Demographics		x				
Medication form		x	x	x	x	Atherosclerosis Risk in Communities form [72]
Satisfaction With Life Scale		x	x	x	x	Quality of life [73]
Physical Performance Tests/Knee Exam						
Height and weight	x	x				To determine BMI
Adverse Events		x	x	x	x	Also collected as they occur
Knee exam		x				To determine eligibility
Pain Severity			x	x	x	To determine MCID [74]
6 min walk		x	x	x	x	Measure of mobility [75]
Expanded Short Physical Performance Battery (SPPB)		x	x	x	x	Gait speed, sit to stand, balance tests; predicts disability [76]
Functional Leg Strength		x	x	x	x	Sit to stand test, part of SPPB
Blood Pressure		x	x	x	x	Overall health measure
GAITRite gait analysis		x	x	x	x	Temporospatial gait variables
Stair climb		x	x	x	x	Timed Stair ascent and descent [77]

*xc* brief screen by self-report, *PSV* Prescreening Visit, *SV* screening visit, *FU* follow-up 6, 12, 18 months after screening visit, *MCID* minimally clinically important difference

#### Screening

Self-reported pain (primary outcome) and physical function are measured using the Likert version of the WOMAC [43]. The pain index (included as a subscale of the KOOS questionnaire [44]) assesses participants’ pain on a scale ranging from 0 (none) to 4 (extreme). The pain subscale consists of 5 items and total scores can range from 0 to 20, with higher scores indicating greater pain. This instrument is recommended by the Osteoarthritis Research Society International as the health status measure of choice for older adults with knee OA. It has been validated for use in orthopaedic and pharmacologic interventions. The pain subscale is also used as a screening tool during SV1 (pain must be ≥ 1 on WOMAC pain subscale). It is also administered to the eligible participants at each scheduled follow-up visit.

For physical function, the Likert version asks participants to indicate on the same scale from 0 (none) to 4 (extreme) the degree of difficulty experienced performing activities of daily living in the last 48 h due to knee pain. Individual scores for the 17 items are totaled to generate a summary score that can range from 0 to 68, with higher scores indicating poorer function.

### Statistical considerations

#### Data management

The Data Management Group of the Coordinating Center has primary responsibility for randomization, quality control, and analyses of data generated by the clinical centers. Primary outcomes and nutrient intake data are collected on hard-copy forms at each site and transferred to an electronic database, while secondary outcomes are collected either electronically or on paper. Our web-based management system assures integrity and validity. Dynamic reports and periodic statistical analyses monitor quality. A participant-based inventory system tracks recruitment, retention, adherence, and missing data from entry through exit, close-out, and lock-down of final datasets.

#### Statistical analyses

All primary analyses are based on intention-to-treat principles in full accordance with CONSORT guidelines [45].

#### Primary analysis

The primary hypothesis of less WOMAC pain in the D + E group at FU18 compared to the N + H attention control group is tested based on a two-tailed significance level of 0.05 using contrast statements from a repeated measures mixed linear model with time (6, 18 months), randomization arm (D + E vs N + H), and the interaction, which adjusts the means at each time point for potential missing data bias. Intervention-effect estimates are further adjusted for baseline pain values, BMI, county, and gender; analysis matches the design, so the variance estimate is not biased.

#### Secondary aim 2

Repeated measures mixed linear models similar to those for Aim 1 are used to analyze WOMAC function, 6-min walk, and SF-36 physical subscale. Each outcome is modeled separately, and 18-month effectiveness tested based on a two-tailed significance level of 0.05.

### Sample size and power calculations

#### Primary outcome

Based on ADAPT, the control group is expected to reduce pain by approximately 10%; therefore, a 15% between-group difference would require a 25% within-group improvement from baseline, exceeding a minimally clinically important improvement (MCII) of 20% [9, 46]. A total sample of 820 (410/group) will provide 94% statistical power to detect differences ≥15% in pain at the 2-sided 0.05 significance level with 80% retention (2-sample *t*-test, Nquery Advisor). In IDEA, the intent-to-treat analysis revealed a 51% reduction in pain in the D + E group, which suggests these assumptions are conservative.

#### Secondary outcomes

Our sample size can detect a moderate effect size of 0.234 at 85% power with relevant detectable differences. However, all estimates from IDEA and ADAPT were collected under rigorously controlled conditions; therefore, the estimates for the pragmatic trial are conservative. We assume a total baseline sample size of N = 820, 80% retention at 18 months, and a 0.05 level of significance for all tests.

### Economic evaluation

To establish the effectiveness of the pragmatic weight loss and exercise intervention appropriate data are collected to address the question of cost-effectiveness. In addition to formal cost-effectiveness analysis, a budget impact analysis will quantify the financial consequences of adopting the program by various payer models, including insurance organizations, healthcare systems, and government, given real-life resource constraints. The results can be used for budget planning and changes in health insurance premiums.

Data on resource use are collected at 6-month intervals. Direct medical costs include costs of inpatient stays and procedures (including knee surgery); outpatient physician, physical therapy, and emergency room visits; laboratory studies, medical devices, and medications, prescribed and non-prescribed. Costs of the intervention includes the meal replacements, monitoring, the wages of intervention and control personnel over the period of the intervention, and any facility rental costs needed to deliver the intervention. Direct nonmedical costs and indirect costs include transportation to intervention centers and other costs to the participant and family to ensure he or she can take part in all **we-can** activities.

We also gather data on indirect costs of lost wages for employed participants (e.g., equivalent to 31% of lost wages in IDEA). Data on time costs (productivity losses) are collected and included in sensitivity analyses. The primary analysis is conducted without indirect costs per recommendations of the Panel on Cost-Effectiveness in Health and Medicine [47, 48]. Quality-adjusted life years (QALYs) is our measure of effectiveness [49, 50]. In all analyses, a 3% per year discounting is applied to both costs and effectiveness [48]. The cost-effectiveness analysis is performed for the trial’s duration and remaining lifetime of the participants.

## Discussion

Exercise and weight loss have level 1 evidence of efficacy [6] for pain relief and the combination is most efficacious [9, 10], yet health practitioners often do not refer patients to community programs, either because they don’t exist or because non-pharmacologic alternatives are not part of the treatment plan [2]. Intensive weight loss combined with exercise reduces abnormal stress by decreasing knee joint loads and reduces abnormal physiology by lowering inflammation [10]. This results in less pain and less disability. Losing 10% of baseline body weight is necessary for a moderate to large clinical effect. We found a dose response to weight change with pain and function, independent of group assignment; IDEA participants who lost more than 10% of baseline body weight had greater reductions in pain and improvements in function than participants who lost between 5 and 10% and less than 5% of baseline body weight [10]. Hence, our minimum weight loss goal for the **we-can** diet and exercise group is 10% of baseline body weight.

Losing weight and maintaining weight loss are both difficult and often unsuccessful. Biological changes fight attempts to lose weight; the body acts in starvation mode increasing feelings of hunger, it suppresses satiety, slows metabolic rate, and attempts to defend higher body weights [51]. We exceeded our mean weight loss goal of 10% in IDEA, in part because participants received regular attention. A similar pragmatic, community based trial of diet and exercise for obese women free of knee OA found that limited patient contact (≤4 h/year) during the 2.5 year intervention period resulted in less than 20% of the D + E group achieving their weight loss goal of ≥ 5 kg or ≥ 5% of baseline body weight (mean weight loss < 1 kg) [52, 53]. Indeed, a possible consequence of a pragmatic approach with little or no participant contact is low adherence. The challenge in **we-can** is to maintain the regular patient contact utilized in IDEA in a less structured, community-based environment. The use of multiple communication techniques with participants is critical in achieving compliance and retention rates that approach those achieved in IDEA. These include, but are not limited to, phone calls, text messages, email, Facebook, LinkedIn, Skype, Twitter, and YouTube. Participants will select their preferred method of communication.

Maintaining intervention fidelity is critical. Fostering close relationships with the community centers that serve as intervention sites is important in maintaining an open line of communication. Site interventionists maintain personal relationships with participants both face to face and electronically. The Coordinating Center staff monitors the quality of the intervention being delivered with periodic face-to-face observations, biweekly Adherence and Retention Committee meetings, and monthly Nutrition and Exercise Committee meetings. Including underserved rural communities such as Haywood and Johnston counties in knee OA clinical trials is unique; however, recruitment and retention may be difficult as communication and transportation concerns are complicated by lower socioeconomic conditions and a less densely populated recruitment base. The recruitment goals for Johnston and Haywood counties have been adjusted to reflect these anticipated impediments.

To meet the chronic disease burden the Centers for Disease Control and Prevention launched several strategic initiatives of which one is to establish community-based programs to support healthy behaviors [22]. Indeed, the physician’s dilemma is the lack of practical means to implement and sustain a diet and exercise program in a community-based environment. **we-can** is designed to test the effectiveness of a community program that will serve as a blueprint and exemplar for clinicians and public health officials in urban and rural communities to implement a weight loss and exercise program designed to reduce knee pain and improve other clinical outcomes in overweight and obese adults with knee OA. We envision a national implementation of this systematic program that will serve as a model for healthcare professionals on implementing a platform that is accessible to consumers and clinicians, and is of value to insurers because it can be sustained long-term and at a reasonable cost.
